# Long-term survival after an aggressive surgical resection and chemotherapy for stage IV pulmonary giant cell carcinoma

**DOI:** 10.1186/1477-7819-3-32

**Published:** 2005-06-02

**Authors:** Fumihiro Shoji, Riichiroh Maruyama, Tatsuro Okamoto, Jiro Ikeda, Tomomi Nakamura, Hiroshi Wataya, Yukito Ichinose

**Affiliations:** 1 Department of Thoracic Oncology, Kyushu Cancer Center, 3-1-1, Notame, Minami-ku, Fukuoka 811-1395, Japan

## Abstract

**Background:**

Pulmonary giant cell carcinoma is one of the rare histological subtypes with pleomorphic, sarcomatoid or sarcomatous elements. The prognosis of patients with this tumor tends to be poor, because surgery, irradiation and chemotherapy are not usually effective.

**Case presentation:**

We herein report a patient with pulmonary giant cell carcinoma with stage IV disease in whom aggressive multi-modality therapy resulted in a long-term survival. A 51-year-old male underwent an emergent operation with a partial resection of small intestinal metastases due to bleeding from the tumor. The patient also underwent a left pneumonectomy due to hemothorax as a result of the rapid growth of the primary tumor. Thereafter, two different regimens of chemotherapy and a partial resection for other site of small intestinal metastases and a splenectomy for splenic metastases were performed. The patient is presently doing well without any evidence of recurrence for 3 years after the initial operation.

**Conclusion:**

This is a first report of a rare case with stage IV pulmonary giant cell carcinoma who has survived long-term after undergoing aggressive surgical treatment and chemotherapy.

## Background

The recent World Health Organization (WHO) classification of lung tumors has unified the heterogeneous group of non-small cell lung carcinomas that contains sarcoma or sarcoma-like components under the designation of "carcinomas with pleomorphic, sarcomatoid or sarcomatous elements" [1]. This group includes different entities, such as pleomorphic carcinoma, spindle cell carcinoma, giant cell carcinoma, carcinosarcoma and pulmonary blastoma. In general, these tumors are rare, comprising approximately from 0.1–0.4% of all lung malignancies [2-4]. The patients with these tumors tend to demonstrate a despondent clinical course and the prognosis for them is gernally poor [5], because surgery, irradiation and chemotherapy are ineffective. We experienced a pulmonary giant cell carcinoma patient with stage IV disease in whom aggressive multi-modality therapy consisting of surgical resections for the primary lesion and multi-organ metastases and also chemotherapy which together resulted in a long-term survival.

## Case presentation

**Figure 1:**
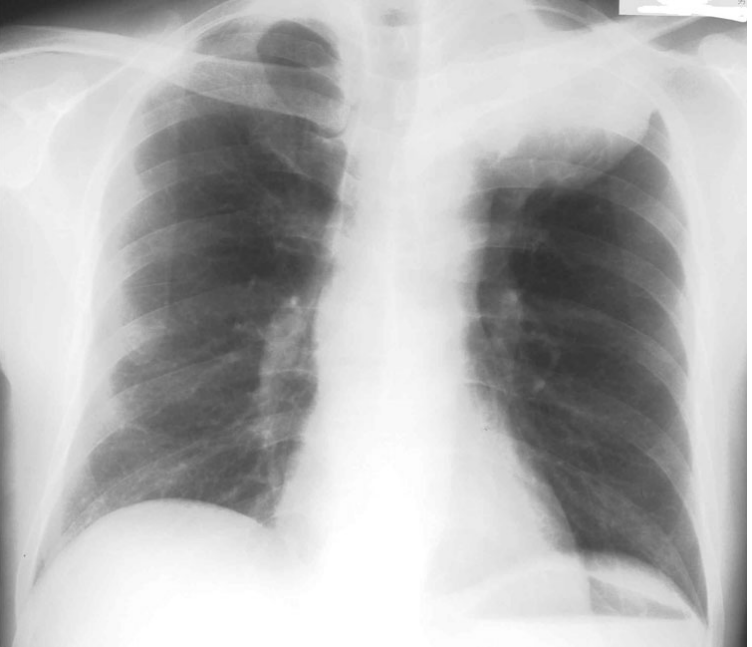
Posterior-anterior view of a chest X-ray film demonstrated a huge mass shadow in the left upper lung field.

**Figure 2:**
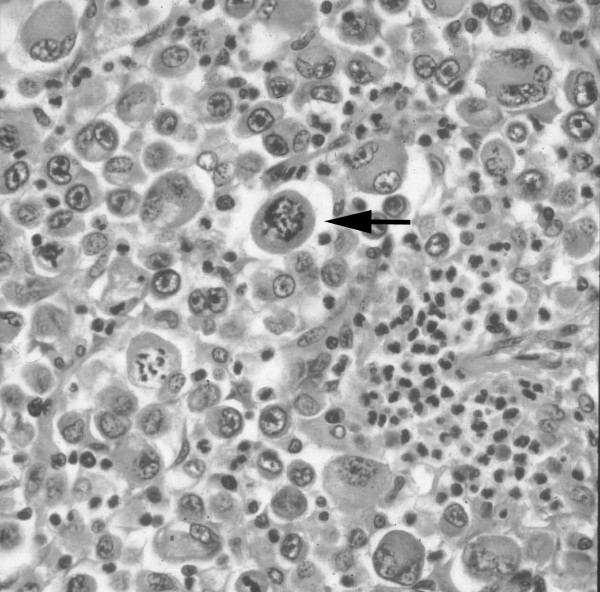
Pathological findings of the left lung. The section consists of a diffuse proliferation of atypical, giant and bizarre cells (arrowhead). No sarcomatoid component is seen.

**Figure 3:**
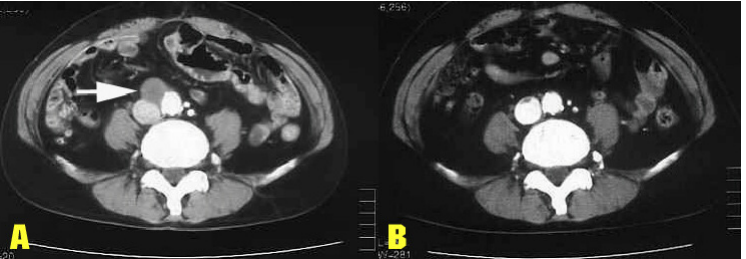
Computerised tomographic scan before and after treatment. A) Abdominal CT showed multiple lymph node swelling, suggesting the presence of metastases (arrowhead). B) Abdominal CT showed the lymph nodes metastases to have completely disappeared.

A 51-year-old male was admitted in June 2001, due to hemosputum, cough, hemo-stool and an abnormal shadow on a chest roentgenogram. Laboratory results showed severe anemia with hemoglobin of 4.0 g/dl (13.6 < normal range < 16.8 g/dl) and hematocrit of 16.0 % (40 < normal range < 48 %). The patient's chest X-ray demonstrated a huge mass lesion in the left upper lung field (Figure 1). Computed tomography (CT) of the chest showed a mass shadow, measuring 7.0 × 7.0 cm in size in the left upper lobe (S^1+2^) without any invasion of the surrounding tissue such as the vessels, plexus or thoracic wall and with no mediastinal lymph node swelling. Abdominal CT revealed a huge mass, measuring 12.7 × 7.5 cm in size in the small intestine. Prior to performing any treatment for the presumed lung cancer, we tried to stop the continuous bleeding from tumor in the small intestine. As a result, we performed an emergency operation. The tumor was observed in the jejunum at a location about 30 cm from the ligament of Treitz on the anal side and a 25 cm length of the jejunum, including the tumor, was thus resected. Six days later, the patient experienced sudden chest pain, dyspnoea and hemoptysis. The patient's chest X ray showed the left lung mass shadow to have rapidly increased in size, while the broncho-fiberscopy findings showed bleeding from the left upper bronchus and an obstruction of the left lower bronchus due to coagulation. Hemothorax due to a rupture of the lung induced by the rapid growth of the tumor was found after an emergency thoracotmy. The tumor was so large that it was difficult to approach the interlobular pulmonary artery. Therefore, a left pneumonectomy with mediastinal lymph nodal dissection was performed. Thereafter, intraoperative intrapleural hypotonic cisplatin treatment [6] was performed because some tumor cells were suspected to exist in the pleural cavity due to the rupture of the tumor. A histological examination revealed pure giant cell carcinoma containing no sarcomatoid component, similar to that found in the small intestine (Figure 2). As a result, we diagnosed the patient to have stage IV disease (pathological stage T2N0M1) according to the TNM classification [1]. The patient had an uneventful recovery without any complications. However, about 4 months after the first operation, the patient was diagnosed to have a recurrence at another site in the small intestine and spleen by abdominal CT. The patient received 2 cycles of chemotherapy (cisplatin 40 mg/m^2 ^+ gemcitabine 800 mg/m^2^+ vinorelbine 20 mg/m^2^), at days 1 and 8, and thereafter every 4 weeks). The splenic metastases increased in size while the size of the tumor in the small intestine decreased. At this time, no recurrence site except for those in the small intestine and spleen were found, therefore, to avoid the risk of bleeding either from tumors in the small intestine or a rupture of spleen in the future, surgical treatment consisting of a partial resection of the small intestine and a splenectomy was performed. The intestinal tumor was found in the jejunum at a location about 10 cm from the ligament of Treitz on the anal side and a total 20 cm length of the jejunum, including the tumor, was resected. A pathological examination revealed a proliferation of pure giant cell carcinoma with extensive necrosis both in the small intestine and the spleen, thus suggesting the chemotherapy to be effective in the both organs. Thereafter, the patient received 2 additional cycles of this triplet chemotherapy. The patient experienced neither any hematological nor severe non-hematological adverse events. About 6 months later, metastases in multiple abdominal lymph nodes were found (Figure 3A). The patient was started on chemotherapy (carboplatin AUC = 2 + paclitaxel 60 mg/m^2^, on days 1 and 8, and thereafter every 3 weeks). After receiving a total of 10 cycles of chemotherapy on an outpatient basis, abdominal CT showed the chemotherapeutic effect to be a complete response (Figure 3B), without any severe hematological or non-hematological adverse events. At present, the patient has survived for about 3-years since the first operation and a complete response has been maintained for 15 months.

## Discussion

According to the treatment guidelines for unresectable non-small cell lung cancer of American Society of Clinical Oncology (ASCO)[7], chemotherapy prolongs survival and is the most appropriate treatment for stage IV patients with a good performance status. Although both resections of primary lung cancer and either brain or adrenal metastases are occasionally recommended in highly selected patients, a surgical resection of other metastasized sites is hardly ever performed. Therefore, the present patient is an extremely rare case because he underwent an emergency surgical resection of small intestinal metastases and a primary tumor due to bleeding from both tumors, as well as a surgical resection of other metastases in the small intestine and spleen in order to avoid a risk of bleeding from the recurrent site in the future.

Fishback *et al*, reported the overall survival of total 78 patients with pleomorphic (spindle/ giant cell) carcinoma (stage I-IV), among whom 57 patients received a surgical resection, to be poor with a median survival time of 10 months and a survival rate of 10% at 5 years [8]. According to Chang *et al*, the mean survival time of resected pleomorphic carcinoma patients was 5 months while the median survival time of pleomorphic carcinoma patients treated with concurrent or sequential chemo-radiotherapy was 2.7 months [9]. To our knowledge, a case of a long-term survivor with stage IV pleomorphic (spindle/ giant cell) carcinoma has never been previously reported. The tumor histology of the present case was very rare, pure giant cell carcinoma, which belongs to the category of carcinomas with pleomorphic, sarcomatoid or sarcomatous elements according to new WHO classification, and the prognosis is estimated to be poor. Although pleomorphic carcinoma has been reported to usually be resistant to chemotherapy, we first chose chemotherapy including cisplatin, gemcitabine and vinorelbine, which has been shown to demonstrate the highest response rate in advanced non-small cell lung cancer based on our experience. In our prior phase II trial using this combination chemotherapy in 79 advanced non-small cell lung cancer patients, the response rate was 56% and the 1-year survival rate was 75% while the toxicity levels were acceptable [10]. After recurrence, we chose chemotherapy with carboplatin and paclitaxel, which is most frequently used for the treatment of advanced non-small cell lung cancer. Since the standard treatment method using carboplatin and paclitaxel in Japan is the administration of AUC of 6 and 200 mg/m^2^, respectively once every 3 weeks [11], the administered regimen (carboplatin AUC = 2 and paclitaxel 60 mg/m^2^, on days 1 and 8, and thereafter every 3 weeks) in this patient was unusual and the dose intensity was relatively small. However, this regimen nevertheless effectively treated his disease and he was also able to work normally during the treatment process. At present, the patient has survived for 3 years since the first operation and has remained healthy without any signs of recurrence for 15 months after the last treatment.

## Conclusion

This is a first report of a rare case with stage IV pulmonary giant cell carcinoma who has survived long-term after undergoing aggressive surgical treatment and chemotherapy.

## Competing interests

The author(s) declare that they have no competing interests.

## Authors' contributions

**FS**: Conceived the study, participated in its design and coordination and drafted the manuscript.

**RM **and **TO**: carried out the literature search and helped in drafting the manuscript

**JI, TN **and **HW**: participated in the study design and helped with preparation of the manuscript

**YI**: Shaped the idea for the manuscript, coordinated the study and edited the manuscript.

All authors conceived of the study, and participated in its design and coordination. All authors read and approved the final manuscript.
